# Evolutionary and Structural Overview of Human Picornavirus Capsid Antibody Evasion

**DOI:** 10.3389/fcimb.2019.00283

**Published:** 2019-08-20

**Authors:** Javier Orlando Cifuente, Gonzalo Moratorio

**Affiliations:** ^1^Structural Biology Unit, Center for Cooperative Research CIC bioGUNE, Derio, Spain; ^2^Laboratorio de Virología Molecular, Centro de Investigaciones Nucleares, Facultad de Ciencias, Universidad de la República, Montevideo, Uruguay; ^3^Laboratorio de Inmunovirología, Institut Pasteur de Montevideo, Montevideo, Uruguay

**Keywords:** picornavirus, capsid, antibody, genetic variability, structure, vaccine

## Abstract

Picornaviruses constitute one of the most relevant viral groups according to their impact on human and animal health. Etiologic agents of a broad spectrum of illnesses with a clinical presentation that ranges from asymptomatic to fatal disease, they have been the cause of uncountable epidemics throughout history. Picornaviruses are small naked RNA-positive single-stranded viruses that include some of the most important pillars in the development of virology, comprising poliovirus, rhinovirus, and hepatitis A virus. Picornavirus infectious particles use the fecal–oral or respiratory routes as primary modes of transmission. In this regard, successful viral spread relies on the capability of viral capsids to (i) shelter the viral genome, (ii) display molecular determinants for cell receptor recognition, (iii) facilitate efficient genome delivery, and (iv) escape from the immune system. Importantly, picornaviruses display a substantial amount of genetic variability driven by both mutation and recombination. Therefore, the outcome of their replication results in the emergence of a genetically diverse cloud of individuals presenting phenotypic variance. The host humoral response against the capsid protein represents the most active immune pressure and primary weapon to control the infection. Since the preservation of the capsid function is deeply rooted in the virus evolutionary dynamics, here we review the current structural evidence focused on capsid antibody evasion mechanisms from that perspective.

## Picornavirus Historical Relevance

Picornaviruses have been pivotal in the foundations of virology. Original research on “ultra-filterable infectious agents” such as foot-and-mouth disease virus (FMDV) and poliovirus (PV) began the era of animal virology (Loeffler and Frosch, 1898; Eggers, 1999). The development of cell cultures for PV replication led to Salk's inactivated and Sabin's attenuated vaccines (Enders et al., 1949). The first animal virus engineered into an infectious clone (Racaniello and Baltimore, 1981) and the first virus synthesized outside the cell was PV (Molla et al., 1991).

Although vast knowledge has been gained, picornaviruses still challenge our understanding. The still open fundamental questions and public health challenges picornaviruses pose reflect that we are far from a conclusive comprehension (Holm-Hansen et al., 2016; Li et al., 2017; Zarocostas, 2018). In the following review, we examine how these agents evade host antibodies (Abs) based on their biological and evolutionary properties, with the spotlight on human picornaviruses.

## Classification and Clinical Impact on Human Health

*Picornaviridae* is a large family of vertebrate viruses that produce both clinically asymptomatic infections but often mild and fatal disease. Their current classification includes more than 30 genera and 75 species (Zell et al., 2017), including several human picornaviruses. The genus *Enterovirus* comprises seven species infecting humans (enterovirus A-to-D and rhinovirus A-to-C). This genus contains poliovirus (PV), coxsackieviruses A/B (CVA/B), enteroviruses (EV), echoviruses (E), and rhinoviruses (RV). Further serological classification results in hundreds of serotypes. Hepatitis A virus (HAV) is the sole human-virus species in the genus *Hepatovirus*. Other human picornaviruses include members of the genera *Cardiovirus* (Saffold virus—SAFV), *Cosavirus* (CoSV), *Parechovirus* (Ljungan virus—LV), *Kubovirus* (Aichi virus—AiV), and *Salivirus* (Salivirus A—SaVA) (Nielsen et al., 2013).

RVs are airborne pathogens, while other enteroviruses and HAV use the fecal–oral route (Yin-Murphy and Almond, 1996). RVs cause the common cold, the most prevalent infectious disease worldwide, resulting in uncountable lost days from school and work. Epidemics occur yearly with outbreaks in the winter and spring (Drysdale et al., 2017). PV infection targets the central nervous system, destroying nerves and motor neurons, resulting in paralytic poliomyelitis. Until the PV worldwide eradication program based on global vaccination, polio epidemics have been the cause of high morbimortality (Minor, 2014). The so-called “non-polio enteroviruses” (CVs, EVs, and Es) cause several diseases with high morbimortality including meningitis, myocarditis, poliomyelitis-like syndrome, pancreatitis, and possibly the onset of diabetes. Outbreaks are common and have been considered to have pandemic potential (Zhang et al., 2015; Pons-Salort et al., 2018). Hepatitis produced by HAV is a mild disease producing liver damage usually leading to total recovery, but rare, severe cases are fatal in older age individuals. HAV produces large outbreaks probably due to its long 2 to 3 week incubation time (Jacobsen and Wiersma, 2010).

## Genome Organization

**Figure 1:**
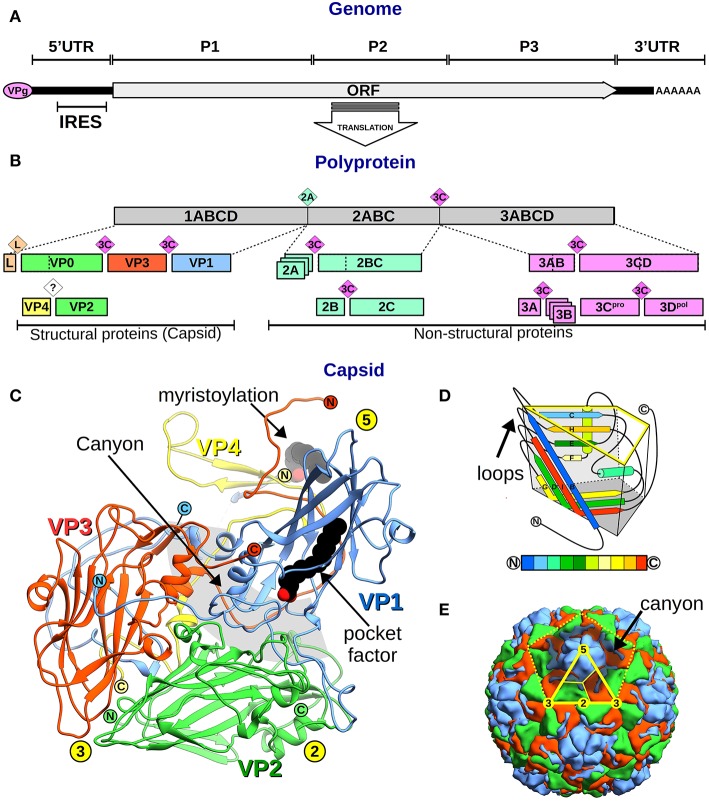
Picornavirus genome, proteins, and capsid organization. **(A)** Representation of the picornavirus genome, the VPg, and the polyA tail, showing the single ORF location. The position of the P1–3 regions, the flanking 5′ and 3′UTR, and the IRES are indicated. **(B)** A bar diagram showing the polyprotein (gray box) and the proteolytic cascade that leads to all picornaviral proteins (colored boxes). Boxes include the protein names following the genome-ORF regions' nomenclature (number–letters) or the VP1–4 nomenclature for the structural proteins. Colored rhombi indicate cleavage points and are labeled with the corresponding protease name. **(C)** Overall view of the canonical picornavirus protomer with the proteins VP1 (blue), VP2 (green), VP3 (red), and VP4 (yellow). The protein N- and C-termini are indicated as encircled N and C letters, and yellow circles show the 5-,−3, 2-fold symmetry axes positions. Lipid components as the VP4 myristoylation and the “pocket factor” are depicted as black spheres. The “canyon” region is shown as a gray circular segment shadow. **(D)** Schematics of the “jelly roll” fold of VP1–3 proteins inscribed in a trapezoidal prism where the yellow highlighted face corresponds to the external capsid surface, and the dark gray base faces the inner capsid. The secondary structure elements are colored from N- to C-terminus according to the color code bar below. External loops and N- and C-terminus are indicated. **(E)** Overall view of the picornavirus capsid showing the outer surface of VP1 (blue), VP2 (green), and VP3 (red). The yellow dotted line indicates the boundaries of one pentamer. The solid yellow line marks the icosahedral asymmetric subunit and thinner lines separate proteins following the trapezoidal schematics shown in **(D)**. Symmetry 5-, 3-, 2-fold symmetry axes are indicated in yellow circles.

Picornaviruses have single-stranded RNA positive-sense genomes (~7–9 kb) that serve as mRNA for viral protein synthesis (Baltimore, 1971) (Figure 1A). Their RNA holds a single ORF encoding a polyprotein precursor for all viral proteins. Importantly, the 5′-end of the genome is covalently bound to the Viral-Protein-genome (VPg) (Crawford and Baltimore, 1983), the primer for viral RNA synthesis (Nomoto et al., 1977). Two untranslated regions (UTR), 5′UTR and 3′UTR, flank the ORF and contain virus-specific RNA secondary structural elements implicated in replication and providing host specificity (Kloc et al., 2018). The 5′UTR bears an internal ribosomal entry site (IRES) and polypyrimidine tract (PPT) that elicit host ribosomes and PPT-binding protein for translation (Martinez-Salas et al., 2018). The 3′UTR finishes in a poly(A) tail that mimics host mRNA tail conferring genome stability.

**Figure 2:**
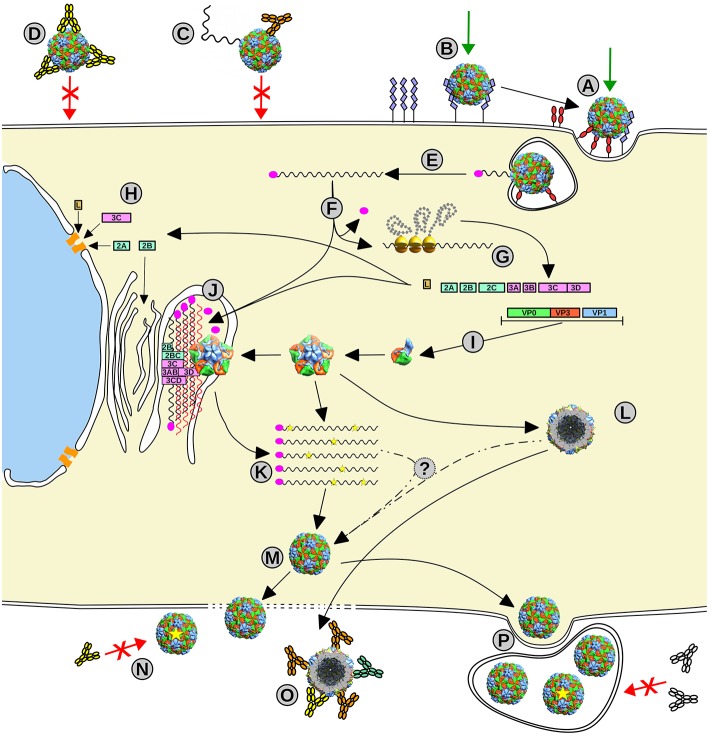
Picornavirus life cycle. (A, B) Picornavirus uses different receptors to enter the cell, some implicated in the signaling internalization (A), meanwhile others can act as carriers that transport the viral particle to meet the primary receptor (B). (C, D) This infection event can be impeded by the action of specific neutralizing antibodies that can destabilize the viral particle (C) or opsonize or stabilize the particle to impair receptor binding or conformational changes required for infection (D). (E) Once the virus enters the cell, the viral RNA delivery mechanism is triggered, and the viral genome (black wavy line) is released into the cytoplasm. (F) Upon removal of VPg (magenta oval), the genome starts the IRES-driven translation leading to the production of the viral polyprotein. (G) The proteolytic cascade produces all viral proteins, structural and non-structural. (H) Some proteins act by hijacking the host cellular systems such as the nuclear pore, the cell translation machinery, and the apoptotic systems and initiate the remodeling of the internal cell membranes. (I) The structural proteins assemble into the capsid intermediates, the protomer and the pentamer, and also procapsids (L). (J) The formed replication complex assembled from non-structural proteins and modified internal membranes firing the picornaviral genome replication by the 3D polymerase *via* RNA complementary (red wavy lines) and using VPg as a primer. (K) The new progeny genomes including eventual mutations (yellow stars). (M) Mature virions assemble from pentamers that surround and package the new viral genomes. Viral particles escape from the cell by cell lysis or budding within membranes that can protect the viral progeny (P). (N) Some progeny virus with mutations in their capsids (yellow star) may escape from to the action of specific NAbs. (O) Empty capsids can act as molecular decoys for Abs to protect the infecting particles from neutralization.

From 5′-to-3′, the ORF comprises three regions: (i) P1, encoding the structural capsid viral proteins (VP4–VP2–VP3–VP1), while, in some picornaviruses, also codifying a short leader L-protein; (ii) P2, encoding the viral non-structural proteins 2A−2B−2C; and (iii) P3 encoding the viral non-structural proteins 3A−3B−3C−3D (Palmenberg, 1990) (Figure 1B). The non-structural proteins' central role is replication, translation, and hijacking host-cell machinery (Figure 2). In particular, 3D is the RNA-dependent RNA polymerase (RdRp or 3Dpol) that synthesizes the virus genome and 3B (which is VPg) acts as its primer, being the only non-structural protein in the virion (Palmenberg, 1990).

## Picornavirus Capsid Anatomy

Picornaviruses were the first human viruses to be structurally defined at the atomic level (Rossmann et al., 1985). To date, several structures of human picornaviruses have been unveiled including HRV, PV, HAV, CVB, E, CVA, EV, SAFV, and AiV (Hogle et al., 1985; Rossmann et al., 1985; Filman et al., 1989, 1998; Muckelbauer et al., 1995; Zhao et al., 1996; Lentz et al., 1997; Hendry et al., 1999; Verdaguer et al., 2000, 2003; Stuart et al., 2002; Zhang et al., 2004; Venkataraman et al., 2008; Plevka et al., 2010, 2012; Zocher et al., 2014; Liu et al., 2015, 2016; Ren et al., 2015; Zhu et al., 2015; Mullapudi et al., 2016). Moreover, the structure of FMDV has been disclosed (Logan et al., 1993; Lea et al., 1994). All picornaviruses have a naked 30 nm icosahedral capsid composed of 60 identical tightly packed protomers (Figure 1E). Early in particle morphogenesis, immature protomers contain VP1 and VP3 together with VP0, the precursor of VP4 and VP2 (Jiang et al., 2014) (Figure 1B). Virus assembly likely goes through a dodecahedral pathway (Li et al., 2012), by the association of pentamers formed by five immature protomers leading to the icosahedral particle, defining 5-, 3-, and 2-fold symmetry axes. Upon genome encapsidation, VP0 is generally auto-catalytically cleaved into VP2 and VP4 generating the mature capsid (except for *Parechovirus* and *Kubovirus*) (Figure 2).

The larger proteins (VP1–3) form the external and internal capsid surface. These proteins have a common fold, the “jelly-roll,” formed by two 4-strand anti-parallel β-sheets and two helices (Figure 1D). Conversely, the small VP4 is located inside the capsid only and usually appears myristoylated at its N-terminus (Paul and Schultz, 1987; Belsham et al., 1991) (Figure 1C). The capsid external surface displays a rugged topography. Main surface features include (i) a principal protrusion built by the interaction of copies of VP1 forming a star-shaped 5-fold vertex, (ii) a 5-fold surrounding valley called the “canyon,” (iii) a VP2 loop protuberance or the “puff,” (iv) a VP3 loop rise or the “knob,” and (v) a large 2-fold depression (Muckelbauer et al., 1995). Loop differences result in distinctive surface traits between picornaviruses. Finally, some picornaviruses exhibit a lipid molecule, the “pocket factor,” bound in a cavity located inside the VP1 jelly-roll, which has been observed to play a role in particle stability (Figure 1C).

## Receptors and Tropism

Picornavirus cell infection starts with its attachment to cell receptors (Figure 2). Therefore, virus-receptor usage is critical for tropism and its evolution can change virus targets at the level of cells to host ranges. The capsid binding sites of picornavirus receptors can be used to classify them into canyon binders and non-canyon binders.

Canyon binders are members of the immunoglobulin superfamily including: (i) ICAM-1 used by HRV and CVA (Greve et al., 1989; Staunton et al., 1989; Tomassini et al., 1989; Kolatkar et al., 1999; Xiao et al., 2001; Baggen et al., 2018), (ii) the PV receptor (PVR) (Mendelsohn et al., 1989; He et al., 2000; Strauss et al., 2015), (iii) the coxsackie and adenovirus receptor (CAR) used by CVBs (Bergelson, 1997; He et al., 2001; Organtini et al., 2014; Lee et al., 2016), (iv) α_v_β_3_ and α_v_β_6_ integrin used by some CVAs (Roivainen et al., 1994; Williams et al., 2004; Shakeel et al., 2013), and (v) the α2 subunit of VLA-2 used by E1 and E8 (Bergelson et al., 1992; Xing et al., 2004). Canyon binders' apical domain engages in the binding into the canyon, triggering conformational changes essential for infection, leading to the altered-particle (A-particle) conformational state (Greve et al., 1989; Xing et al., 2004; Xiao et al., 2005; Shakeel et al., 2013; Organtini et al., 2014; Strauss et al., 2015). These changes have been observed to depend on the number of binding events that stimulate the viral particle (Lee et al., 2016). Engagement of several receptors is known to bring the viral 5-fold vertex close to the cell membrane (Bubeck et al., 2005).

Non-canyon binders attach to the virus surface elsewhere outside the canyon, tethering the virus to the cell surface eventually signaling for virus internalization. Importantly, they are diverse in molecular characteristics. These receptors include (i) the LDL-receptor used by HRV-C (Verdaguer et al., 2004); (ii) the decay-accelerating factor (DAF), receptor of many echoviruses and CVBs (Bergelson et al., 1994, 1995; Pettigrew et al., 2006; Plevka et al., 2010; Pan et al., 2011; Yoder et al., 2012); (iii) P-selectin glycoprotein ligand-1 (PSGL-1) used by EV71 (Nishimura et al., 2009); and (iv) scavenger receptor B2 (ScaRB2), a receptor of EV71 (Yamayoshi et al., 2009; Zhou et al., 2018). This group possibly includes heparan sulfate used by several enteroviruses (Escribano-Romero, 2004; Zautner et al., 2006; McLeish et al., 2012; Tan et al., 2013; Nishimura et al., 2015) and cadherin-related family member 3 (CDHR3) used by HRV-C (Watters and Palmenberg, 2018). Non-canyon binders rarely induce substantial conformational changes due to their interaction, and some may not be essential for infection since few mutations allow or limit their usage (Pan et al., 2011; McLeish et al., 2012; Lee et al., 2013). This alternative receptor usage can modify the infection mechanism, as seen in the case of CVB3 binding to DAF which signals the trafficking of the attached virus to tight junctions where the virus can meet CAR (Coyne and Bergelson, 2006).

## Population Dynamics and Genetic Variability

Picornaviruses display a great potential for adaptation and evolution, which is primarily dictated by their high mutation rate. Viral progenies are huge in population size and they have short generation times. Thus, the RNA virus population is a dynamic cloud of mutants where the average-consensus sequence of all variants represents the “genotype.” The mutation rate is the number of genetic changes, such as point mutations, insertions, or deletions introduced during viral replication. The first mutation rate measurement on RNA viruses was reported for CVA, disclosing a value of 1 mutation every 10,000 nucleotides copied (Eggers and Tamm, 1965).

Natural selection may have shaped picornaviral mutation rates in response to extremely dynamic ecosystems (Elena and Sanjuán, 2005). Therefore, this natural low replicative fidelity results in populations that quickly adapt to unexpected changes in the environment, such as immune pressure (Andino and Domingo, 2015). These observations have been conceptualized in the light of quasispecies evolution (Domingo et al., 2012). This adaptive capacity can be impaired by altering viral mutation rates (Vignuzzi et al., 2006). Indeed, the first support of the role of replicative fidelity in viral pathogenesis was observed in picornaviruses. Two groups isolated the first antimutator variant of an RNA virus, by serially passaging PV in the presence of ribavirin (Pfeiffer and Kirkegaard, 2003; Vignuzzi et al., 2006). The resistant variant contained a 3Dpol single point mutant (G64S) relatively resistant to lethal mutagenesis leading to (i) populations with lower mutation rates, (ii) reduced genetic diversity, and (iii) attenuated phenotype in mice (Vignuzzi et al., 2006). Recently, it has been suggested that this attenuation could be partly an outcome of a decrease in the replication speed (Fitzsimmons et al., 2018). Moreover, genetic engineering of viral polymerases has also identified several low-fidelity variants, called mutator variants (Thompson et al., 2007; Gong and Peersen, 2010; Gnädig et al., 2012; Rozen-Gagnon et al., 2014). For instance, using FMDV as a model, low-fidelity variants were found to increase mutation frequencies and render these viruses more susceptible to mutagenesis (Xie et al., 2014). Moreover, the same residue in the 3Dpol of FMDV is responsible to increase or decrease fidelity (Rai et al., 2017). Thus, picornaviral mutator variants were proven to increase mutation frequencies, decrease viral fitness, and also display an attenuated phenotype. Ostensibly, picornavirus mutation rates have been tuned to be near an upper limit (Crotty et al., 2001) yet evading population extinction by the accumulation of deleterious mutations by harmonizing (i) genetic integrity, (ii) genetic diversity, and (iii) replicative speed.

In addition to the classical view of single virus infectious unit of picornaviruses, structures containing many viral genomes support the existence of collective infectious units (Sanjuán, 2017). Lipid vesicles have been observed in HAV and EV infections (Feng et al., 2013; Chen et al., 2015; Kirkegaard, 2017). This current evidence incorporates the vesicle release and transmission to the standard lytic release and transmission of free virions, opening the debate on the “social evolution” of picornaviruses. Social evolution has been proposed to reduce detrimental mutations and negative interactions among the individuals within the population with direct implications for viral evolution, genetic diversity, and viral fitness (Bordería et al., 2015).

## Recombination

During infection, RNA virus genomes can interchange nucleotide sequences resulting in genetic variation by recombination resulting in unpredictable advantages (Simon-Loriere and Holmes, 2011). This phenomenon was discovered in cells co-infected with PV escape mutants, resistant to antisera and guanidine, resulting in recombinant infectious PV (Ledinko, 1963). Recombination is widespread at intra-typic and inter-typic levels (Lukashev, 2005, 2010), often preceding the emergence of novel evolutionary lineages of picornaviruses (McWilliam Leitch et al., 2009, 2010; Meijer et al., 2012). For instance, there is evidence about recombinants of Sabin-related polioviruses harboring homologous sequences of other species of enteroviruses (Arita et al., 2005; Combelas et al., 2011; Bessaud et al., 2016).

Interestingly, recombination-deficient variants of PV have been identified. These viruses carry amino acid changes in the in 3Dpol that reduce recombination without conferring other detectable replication deficiencies. These non-recombining viruses accumulate a higher number of detrimental mutations, presumably by an inability to purge deleterious mutations, and fewer beneficial mutations (Xiao et al., 2016, 2017). Lately, the combined approach of mathematical modeling and experimental evolution experiments have predicted the frequency of recombination of picornaviruses such as PV and EV71 (Stern et al., 2017; Woodman et al., 2018).

## Antibody Response Against Picornavirus Infection

Innate immune response detects foreign RNA using sensing proteins such as RIG-I, MDA-5, and Toll-like Receptor-3. These mediators act in the early control of picornaviral infection (Slater et al., 2010). Nevertheless, the adaptive response mediated by Abs plays the definitive role in the resolution of the infection. Several pieces of evidence support this view as (i) patients with agammaglobulinemia develop chronic infections (Wilfert et al., 1977; McKinney et al., 1987; Kainulainen et al., 2010; Bucciol et al., 2018), (ii) humoral response mediates the protective effect of picornavirus vaccines (Grant et al., 2017; Sun et al., 2017), (iii) mice lacking B-cells cannot clear enteroviral infections (Mena et al., 1999), and (iv) passive immunization with Abs is an efficient treatment of HAV infection (Stapleton, 1992), although their effectiveness for enteroviral neonatal infections is disputed (Yen et al., 2015). Therefore, it appears to be clear that an effective humoral immune response represents the final host weapon to shortcut the viral infection.

## Antibody Escape Mutants

Picornaviruses' high mutation rates permit the rapid escape from the immune system. The change of residues in the exterior capsid surface overcomes the intense pressure exerted by host Abs. These properties were used to locate capsid antigenic sites by testing neutralizing Abs (nAbs) escape mutants *in vitro* (Minor et al., 1986; Sherry et al., 1986; Stapleton and Lemon, 1987). Successful progeny virus rely on capsid functionality. Therefore, the preservation of the architecture and receptor binding restrict viable mutations. The exposed jelly-roll loops can accommodate mutations easier than secondary structure elements and protein–protein interacting surfaces (Murray et al., 1988; Usherwood and Nash, 1995). Several structural studies by cryoEM revealed the way nAbs bind to solvent-exposed loops of VP1–3 by interacting with critical residues. Complexes of viruses with Fab-Abs fragments can display 1-Fab:1-protomer ratio following the icosahedral symmetry. Nevertheless, when epitopes are close to the symmetry axis, lower binding ratios are observed due to Fab–Fab steric hindrance (Lee et al., 2013). Three mechanisms of neutralization are interpreted from these structures: (i) destabilization of the virion by triggering conformational changes upon Fab binding, which is accompanied by the “pocket factor” release when present (Smith et al., 1996; Plevka et al., 2014; Dong et al., 2017; Zheng et al., 2019), (ii) stabilization of virions by cross-linking of protomers to prevent conformational changes for infection (Ye et al., 2016), (iii) virus aggregation by antibody cross-linking particles (Mosser et al., 1989), and (iv) opsonization that can interfere with virus-cell attachment and receptor binding (Lee et al., 2013; Wang et al., 2017).

## The Canyon Hypothesis

Hiding the receptor-interacting surface from Abs surveillance is the hypothetical function of the canyon (Rossmann, 1989). Therefore, the canyon is the result of an evolutionary process to preserve and protect the critical residues required for host-cell receptor recognition. Conversely, accessible areas can mutate to disguise the virus from the humoral immune response. Nevertheless, some observations have challenged this view, proposing the receptor-binding site topology as an uncoating mechanism that dictates receptor binding to trigger the uncoating event (Smith et al., 1996). These views are not strictly incompatible; hence, both pictures contribute to the paradigm of picornavirus capsid evolution. Here, capsid topology arose out of and continues to be shaped by the interplay of host environmental pressure, random genome mutations, and fixation of mutations when beneficial.

## Picornavirus Antibody Decoy Particles

Picornaviruses are known to produce a significant amount of procapsids during the infectious cell cycle, which appears as an inefficient way to replicate (Shingler et al., 2015). This wastefulness looks aggravated considering each polyprotein translation event would lead to a single protomer. Finally, the so-called procapsid may be an off-pathway particle (Cifuente et al., 2013). Although counterintuitive in appearance, the function of the procapsid could be to act as an immune decoy to enhance the infectivity of mature virions providing an evolutive advantage (Shingler et al., 2015; Liu et al., 2016). In this regard, empty Dane particles also have been proposed to be decoy particles for the hepatitis B virus (Rydell et al., 2017).

Several picornavirus procapsids are larger particles compared to the mature virion and similar in shape to the A-particle. Procapsids have some viral epitopes more accessible and consequently can bind nAbs more efficiently. This phenomenon has been observed for the procapsid and mature virion of EV71, revealing that they are antigenically distinct (Shingler et al., 2015). Moreover, a non-nAb has been structurally solved in complex with procapsids but not disclosed for the mature virion (Hewat and Blaas, 2006), which suggests that procapsid may also lead to effects in the modulation of non-nAbs immune response.

## Vaccines

Inactivated vaccines for PV, HAV, and FMDV are shown to prevent associated illnesses by inducing specific antibody defenses (Salk, 1957). Nevertheless, it was the live-attenuated oral PV vaccine (OPV) responsible for most of the success in controlling polio epidemics. PV attenuation was obtained by serial passage in cell lines from non-human hosts (Sabin, 1965). Rare cases of vaccination-derived paralytic disease can occur as well as vaccines shedding of virulent poliovirus revertant (Platt et al., 2014).

New ideas for picornavirus vaccines, currently under development, exploit evolutionary concepts including (i) codon usage, (ii) mutational robustness, (iii) modification of translational efficiency, and (iv) RdRp fidelity manipulation (Burns et al., 2006; Mueller et al., 2006; Coleman et al., 2008; Le Nouën et al., 2014; Tulloch et al., 2014; McDonald et al., 2016). In this regard, synonymous codon deoptimization was first applied for PV (Burns et al., 2006; Mueller et al., 2006) and other picornaviruses such as FMDV (Diaz-San Segundo et al., 2016). Interestingly, increasing the CpG and UpA dinucleotide frequencies using synonymous codon substitutions leads to increased activation of the immune system (Tulloch et al., 2014). The maintenance of phenotype despite changing the genotype (mutations), or mutational robustness, can be altered by codon substitutions, correlating less robust viruses with attenuation in mice (Lauring et al., 2012). This approach was further extended using synonymous codons that upon mutation became stop codons (Moratorio et al., 2017). The increase in stop codon mutations in codon-engineered CVB3 during replication led to a loss of infectivity *in vitro* and attenuation *in vivo*. These new methods can improve the safety of already existing live-attenuated vaccines and can be broadly applied by re-coding any viral genome (Jorge et al., 2015).

Further elucidation of the mechanisms underlying these phenotypes could be used for rational codon rewiring in combination with increasing CpG and UpA frequencies to activate innate host immunity (Kumagai et al., 2008; Tulloch et al., 2014).

## Final Remarks

The control and eradication of pathogenic picornaviruses is an ongoing problem. Picornavirus evolution, ruled by high mutation rates and recombination, has made this viral group genetically and antigenically highly variable. Moreover, changes in virulence represent an unpredictable threat. Important lessons drawn from the polio eradication battle indicate the necessity of a new generation of vaccines (Agol et al., 2016). A holistic approach based on big data and mathematical modeling combining views from structural and evolutionary biology, cellular, and molecular immunology will allow a better understanding of picornavirus–host interactions. This knowledge could have the potential to foresee possible outbreaks and changes on viral virulence. Furthermore, these models may redefine the way new vaccines and antiviral therapies will be designed.

## Author Contributions

JC and GM conceived the review project, designed figures, wrote the manuscirpt, and approved it for publication. They both contributed equally to the work.

### Conflict of Interest Statement

The authors declare that the research was conducted in the absence of any commercial or financial relationships that could be construed as a potential conflict of interest.
